# Differential Susceptibility of Mixed Polymicrobial Biofilms Involving Ocular Coccoid Bacteria (*Staphylococcus aureus* and *S. epidermidis*) and a Filamentous Fungus (*Fusarium solani*) on Ex Vivo Human Corneas

**DOI:** 10.3390/microorganisms11020413

**Published:** 2023-02-06

**Authors:** Sisinthy Shivaji, Banka Nagapriya, Konduri Ranjith

**Affiliations:** Jhaveri Microbiology Centre, Prof. Brien Holden Eye Research Centre, L. V. Prasad Eye Institute, Kallam Anji Reddy Campus, Hyderabad 500034, Telangana, India

**Keywords:** antimicrobial resistance, biofilm, ocular bacteria, ocular, fungi, polymicrobial biofilm, mixed polymicrobial biofilm, *S. epidermidis*, *S. aureus*, *F. solani*

## Abstract

Biofilms confer several advantages to the organisms associated with them, such as increased resistances to antibacterial and antifungal compounds compared to free living cells. Compared to monomicrobial biofilms involving a single microorganism, biofilms composed of microorganisms affiliated to bacterial and fungal kingdoms are predominant in nature. Despite the predominance of polymicrobial biofilms, and more so mixed polymicrobial biofilms, they are rarely studied. The objective of the current study is to evaluate the potential of ocular bacteria and a filamentous fungus to form monomicrobial and mixed polymicrobial biofilms on synthetic and natural substrates and to monitor their response to antibiotics. In this sense, we demonstrated that the ocular pathogens *Staphylococcus aureus*, *S. epidermidis,* and *Fusarium solani* form monomicrobial and mixed polymicrobial biofilms both on tissue culture polystyrene plates and on ex vivo human corneas from cadavers using confocal microscopy and scanning electron microscopy. Additionally, the mixed polymicrobial biofilms involving the above ocular bacteria and a filamentous fungus were less susceptible to different antibacterials and antifungals in relation to the corresponding control planktonic cells. Further, the MICs to the screened antibacterials and antifungals in polymicrobial biofilms involving a bacterium or a fungus was either increased, decreased, or unchanged compared to the corresponding individual bacterial or fungal biofilm. The results would be useful to the ophthalmologist to plan effective treatment regimens for the eye since these are common pathogens of the eye causing keratitis, endophthalmitis, conjunctivitis, etc.

## 1. Introduction

A great proportion of human infections (80%), for example, otitis [1], wound infection in diabetic patients [2], sinusitis [3], cystic fibrosis which augments predisposition for the development of recurrent infection, [4] etc., are associated with bacteria that are involved in the formation of a biofilm. Ocular surface bacteria and fungi also possess the potential to form monomicrobial biofilms [5,6,7,8,9,10,11,12] on prosthetic ocular devices such as intraocular lenses, contact lenses, scleral buckles, etc. [11]. This ability to form biofilms by ocular bacteria has also been implicated in ocular surface diseases such as keratitis [13]. In addition, it has also been demonstrated that in the biofilm phase, the susceptibility to an antimicrobial treatment is altered such that the minimum inhibitory concentration (MIC) of an antimicrobial agent is increased by >100 fold compared to the planktonic phase cells. Such an increase in MIC to antimicrobial agents has also been reported for several ocular bacteria, namely coagulase negative *Staphylococcus* spp., *S. aureus*, *S. epidermidis*, *Serratia marcescens*, *Klebsiella pneumonia*, *Pseudomonas aeruginosa*, *Streptococcus pyogenes*, *S. pneumoniae*, and *E. coli* [12,14,15,16,17]. Thus, the formation of a biofilm is of great relevance to human health, especially with respect to the acquired resistance to routinely used drugs in the clinics.

In nature, polymicrobial biofilms involving multiple species of the same genus or mixed polymicrobial biofilms involving taxa from the bacterial and fungal kingdoms are dominant [18,19]. Polymicrobial biofilms were first described in the oral cavity [20,21] and in chronic wounds [22], but compared to monomicrobial and polymicrobial biofilms, mixed polymicrobial biofilms have rarely been monitored [23,24,25,26] as in dental caries involving *C. albicans* and *S. mutans* [27,28], in chronic wounds involving *P. aeruginosa* and *S. aureus* [29,30,31], and in cystic fibrosis patients involving *Aspergillus fumigatus* and *P. aeruginosa* [32,33,34]. Such mixed polymicrobial biofilms are all the rarer on the ocular surface. In a recent study, we reported that ocular *S. aureus*, *S. epidermidis,* and *C. albicans* [9], formed mixed polymicrobial biofilms on the human cadaveric cornea. Further mixed polymicrobial biofilms involving a filamentous fungus and a bacterium on the ocular surface have never been studied. The reason could be that mixed polymicrobial biofilms involving a bacterium and a filamentous fungus were not attempted until a landmark paper titled “Can filamentous fungi form biofilms?”, where Harding et al. (2009) demonstrated that surface-associated filamentous fungi can form biofilms. Subsequently it was confirmed that filamentous fungi such as *A. fumigatus*, *Aspergillus* spp., *F. graminearum*, *F. solani*, *F. oxysporum*, *Fusarium* spp., *Botrytis* spp., and *Verticillium* spp. [35,36,37,38,39,40,41,42,43] form biofilms. Despite these studies, mixed polymicrobial biofilms of a filamentous fungus and a bacterium on the ocular surface have been rarely studied.

In this study, ocular isolates of *S. aureus*, *S. epidermidis*, and a filamentous fungus (*F. solani*) were used to compare their potential to form monomicrobial (single bacterium or fungi) and a mixed polymicrobial (combination of a bacterium and a fungus) biofilm on human cadaveric cornea. In addition, antimicrobial resistance was also monitored in the above combinations to 18 different antibiotics of different classes and 6 antifungals commonly used in treating diseases of the eye. The results indicated that all the three microorganisms formed biofilms on ex vivo human corneas, the biofilm thickness varied depending on the combination of the microbes involved, and the mixed polymicrobial biofilms were more resistant to antibiotics compared to planktonic cells, but with respect to monomicrobial biofilms, the response was either increased, decreased, or exhibited no change.

## 2. Materials and Methods

### 2.1. Study Centre

The L V Prasad Eye Institute (LVPEI), Hyderabad, India, is an eye care facility recognized as a World Health Organization (WHO) Collaborating Centre for Prevention of Blindness.

### 2.2. Ethics Statement

The research undertaken in this study was approved by the Ethics committee of the LVPEI (LEC-BHR-P-04-21-623). The ocular isolates were a gift obtained from the Jhaveri Microbiology Centre of the same Institute.

### 2.3. Cultivation of the Bacterial Isolates

Purified cultures of ocular *S. aureus* and *S. epidermidis* were maintained on 5% sheep blood agar medium plates [44] and characterized using phenotypic methods and 16S rRNA gene sequencing [12]. On Mannitol salt agar (MSA), isolate L-1054-2019 (2) produced a yellow pigment and was oxidation–fermentation and coagulase test positive, which was suggestive of *S. aureus*. On the other isolate, L-1058-2019 (2), *S. epidermidis* appeared as pink colonies on MSA agar plates and as white opaque colonies on non-hemolytic blood agar and the isolate was negative for the oxidation–fermentation and coagulase test. The identity was also confirmed using Vitek 2 Compact System. The isolates were preserved at −80 °C in tryptone soya broth with 30% glycerol and routinely cultured at 37 °C on 5% sheep blood agar at 37 °C [12].

### 2.4. Cultivation of the Fungal Isolate

*F. solani* (L-1579/2020) formed white, puffy cotton-like colonies and formed chlamydospores when cultured on potato dextrose agar (PDA). Chlamydospores were identified using the Calcofluor White (CFW) staining method. *F. solani* was further identified by sequencing the phylogenetic markers ITS1 and ITS2 [45]. *F. solani* was preserved in TSB [44] and was routinely grown in Sabouraud dextrose medium (SDM) [9].

### 2.5. Biofilm Formationby Ocular Bacteria and Fungi

The formation of a biofilm in the two ocular Staphylococci isolates and F. solani was evaluated by the tissue culture plate method (TCP) using either crystal violet (CV) or XTT [2,3-Bis-(2-methoxy-4-nitro-5-sulfophenyl)-2H-tetrazolium-5-carboxanilide] (Sigma, USA), as described earlier [9,12].

In the CV method, an overnight culture in YPD medium [(bacteriological peptone (20%), glucose (20%) and yeast extract (20%)] was diluted 10,000 times (*v*/*v*) and then 100 μL of the suspension, equivalent to 10^4^ cells/mL, was incubated in a 96-well plate containing 100 μL of fresh YPD medium at 37 °C for 24 h and 48 h. After incubation, the YPD medium was decanted, the planktonic cells discarded, and the cells that adhered to the wells were washed twice with 200 μL of phosphate-buffered saline (PBS, contains 137 mM of NaCl, 2.7 mM of KCl, 10 mM of Na_2_HPO_4_, and 1.8 mM of KH_2_PO_4,_ pH 7.4), and the plates were air-dried at room temperature (RT). The bacterial cells that had adhered to the wells were stained using 0.1% aqueous crystal violet (Sigma Chemical Co., St. Louis, MO, USA) and subsequently the excess CV was decanted, and each well was washed twice with 200 μL of PBS and dried at RT. CV associated with the bacteria was extracted with 200 μL of absolute ethanol. From each well, 100 μL was then transferred to a fresh 96-well plate and the biofilm formation was quantified using a SpectraMax M3 Spectrophotometer (Molecular Devices, San Jose, CA, USA)] set at 595 nm. Wells without cells served as the control (OD was <0.1 at 595 nm) and the OD value was deducted from the “high-biofilm formers” (OD > 0.3 at 595 nm) and “low-biofilm formers” (OD < 0.3 at 595 nm). *S. aureus* ATCC 25922 (positive for biofilm formation) and E. coli ATCC 25923 (negative for biofilm formation) served as the positive and negative controls, respectively, for biofilm formation [7,44,45]. The experiment was performed with three replicates.

In the XTT method, the cells were diluted 10,000 times with YPD medium as in the CV method and then 100 μL of the suspension was incubated in YPD in a 96-well plate for 24 h and 48 h. The media was then decanted and each well was washed twice using 200 μL of autoclaved milliQ water and allowed to air dry for 30 min. The washed cells were stained in the dark with 200 μL of XTT solution [147 μL of PBS and 50.5 μL of XTT (1 mg/ mL, Sigma Chemical Co., St. Louis, MO, USA) and 2.5 μL of Menadione (0.4 mM, Sigma Chemical Co., St. Louis, MO, USA)] and incubated in the dark at 37 °C for 3 h [12,34,46]. From each well, 100 μL was then transferred to a fresh 96-well plate and biofilm formation was quantified using a SpectraMax M3, microplate reader (Molecular Devices, CA, USA) set at 490 nm. The negative and positive controls used were identical to that used for the CV method. All experiments were performed using three replicates.

### 2.6. Monitoring Mixed Polymicrobial Biofilms

Mixed polymicrobial biofilms involving the fungus *F. solani* with the coccoid bacteria *S. aureus* or *S. epidermidis* were generated by co-incubation in YPD broth in tissue culture plates. The mixed polymicrobial biofilms included 10^4^ cells /mL of the bacterium or fungus co-incubated in the following combinations, *S. aureus* and *F. solani* and *S. epidermidis* and *F. solani*, respectively, at 37 °C for 24 or 48 h. After the incubation, the planktonic cells were decanted, washed with 1X PBS, and stained with CV and XTT separately as described above in Section 2.5.

### 2.7. Confocal Laser Scanning Microscopy for Monitoring the Biofilm Thickness on Human Cadaveric Corneas

Human cadaveric corneas were procured from the Ramayamma International Eye Bank (RIEB), LVPEI, Hyderabad, following the approval by the institutional review board and human ethics committee. Only corneas of a low quality and not suitable for corneal transplantation were used. These corneas were washed with PBS and then immersed in plain DMEM medium overnight at 37 °C in a 5% CO_2_ incubator to minimize the residual antibiotics [9,47]. The corneas were oriented with their epithelial surface facing upward and then three horizontal and vertical cuts were created with a sterile steel scalpel [9,47]. Subsequently, an overnight culture of the bacterium and the fungus were in YPD. The broth was diluted with bacteria (10^3^ cells/ mL) and fungi (10^3^ cells/ mL) and centrifuged at 12,000 rpm (Eppendorf, MA, USA, model no: 5430) at 25 °C to obtain a pellet of cells. The pellet was washed again with 200 μL of autoclaved distilled water and pelleted again; it was suspended in 100 μL of DMEM and pipetted onto the corneas and subsequently left undisturbed for 24 or 48 h at 37 °C in a 5% CO_2_ incubator. These corneas were also co-incubated with the bacterium and the fungus for 24 h and 48 h. After incubation, the corneas were then washed with autoclaved distilled water, fixed for 3 h with 250 µL of 4% formaldehyde, and then washed free of the fixative and stained with 1.67 µM of Syto^®^9, a nuclear fluorescent dye (Invitrogen, Carlsbad, CA, USA), for 30 min followed by staining in the dark with 0.025% Calcofluor white M2R [7,9,48]. Syto^®^9 binds to the β-linked polysaccharides of EPS and fluoresces blue under long-wave UV light, whereas calcofluor white binds to the exopolysaccharides (EPSs) involved in the biofilm formation in a variety of organisms. For the visualization of the biofilms by confocal microscopy, Syto9 and Calcofluor were excited at 490 nm and 363 nm, respectively, and the thickness was measured as described earlier [9]. The values representing the Z axis (Average ± standard deviation) in µm and an unpaired T test was used to calculate the significance between the groups (*p*-value < 0.05 was considered significant).

### 2.8. Scanning Electron Microscopy for Visualisation of Biofilms on Human Cadaveric Cornea

The human cadaveric corneas were co-incubated with either the bacterium, fungus, or a combination of bacterium and fungus, as described above, for the confocal laser scanning microscopic studies and processed for scanning electron microscopy (SEM). The corneas following incubation with the microbes were washed, fixed with 2.5% glutaraldehyde, washed once again, serially dehydrated with absolute ethanol, and finally air dried overnight, as described earlier [11]. The biofilms were then sputtered with gold for 60 s and visualized using an SEM (Carl Zeiss Model EVO 18, Carl Zeiss, Germany) operated at 5–20 kV [9,12].

### 2.9. Susceptibility to Antimicrobials in Planktonic Phase

Both the antibacterials and antifungals were evaluated for their activity, as per the standard protocol of the Clinical and Laboratory Standards Institute, using an overnight grown bacterial/fungal suspension as described earlier [8,9,12,46]. The minimum inhibitory concentration (MIC) required for inhibiting the growth of the microorganisms was determined for each of the antibacterials (Aminoglycosides: Amikacin, Gentamicin, and Tobramycin; β-lactam: Ampicillin; Cephalosporins: Cefuroxime, Ceftriaxone, Cefepime, and Cefazolin; Fluoroquinolones: Gatifloxacin, Moxifloxacin, Ciprofloxacin and Ofloxacin; Amphenicols: Chloramphenicol; Macrolide: Azithromycin; Nitroimidazole: Metronidazole; Lincosamide: Clindamycin and Lincomycin; Tetracycline: Monocycline) and the antifungal drugs (Amphotericin B, Caspofungin, Fluconazole, Itraconazole, Natamycin and Voriconazole). The susceptibility tests were determined in triplicate.

### 2.10. Susceptibility to Antimicrobials in Monomicrobial Biofilms

The procedure followed was essentially as described earlier [8,12,46]. Briefly, the bacterium or fungus were allowed to form biofilms as above for 48 h in the wells of tissue culture plates. Subsequently, the medium from the wells was decanted and the wells were washed with PBS and exposed to a known antibacterial/antifungal drug concentration, as mentioned in Section 2.9. After 24 h of incubation, the wells were washed with PBS and processed for monitoring the MIC by the XTT method [8,12,46]. The wells with the cells but without the drug served as a negative control. All the drug treatments were repeated thrice.

### 2.11. Susceptibility to Antimicrobials in Mixed Polymicrobial Biofilms

Mixed polymicrobial biofilms were generated by co-incubating a bacterium plus fungus in YPD to form a biofilm for 48 h as above and were washed and co-incubated for an additional 24 h in the presence of the antimicrobial agent at the desired concentration, as mentioned in Section 2.9. Subsequently, the mixed polymicrobial biofilms were washed and processed for monitoring the MIC by the XTT method [9]. The inoculums incubated without the drug served as a negative control. All drug treatments were repeated thrice.

### 2.12. Statistical Analysis

All comparisons were made using Chi^2^ test. A *p*-value < 0.05 was considered significant.

## 3. Results and Discussion

Polymicrobial biofilms involving multiple bacteria, bacteria and fungi, bacteria and an algae, and bacteria or protozoan [19] are probably more predominant in nature [18]. Such polymicrobial biofilms are also common in health care and have been reported for bacteria residing in the oral cavity [20,21], chronic wounds [22], infections of the lung, inner ear, urinary tract, oral cavity, wounds, and teeth, and those that dwell on medical devices [49,50]. Further, it was observed that the dual species involved in the formation of polymicrobial biofilms were different, for example *S. aureus* and *P. aeruginosa* [51] and *Staphylococcus xylosus* and *S. aureus* [52]. In comparison to polymicrobial biofilms involving two bacteria, mixed polymicrobial biofilms involving bacteria and fungi have been rarely monitored [23,24,25,26] as on endotracheal tubes and urinary tract catheters (involving *C. albicans* and *E. coli* or *S. aureus*) and in the lungs of cystic fibrosis patients (*A. fumigatus* and *P. aeruginosa*) [32,33,34]. In vitro, also mixed polymicrobial biofilms (*C. albicans* and respiratory pathogen *S. aureus*) [53] and clinical strains of *S. aureus* and *C. tropicalis* and *C. tropicalis* and *S. marcescens* [54] have been monitored. To the best of our knowledge, mixed polymicrobial biofilms on the ocular surface have been rarely reported [9].

In the current study, we demonstrated that ocular *S. aureus*, which causes dacryocystitis, conjunctivitis, keratitis, cellulitis, corneal ulcers, and endophthalmitis [55,56], *S. epidermidis*, which causes blepharitis and suppurative keratitis [57], filamentous fungus *F. solani*, which causes keratitis, and endophthalmitis [57] could form mixed polymicrobial biofilms on ex vivo human corneas [9]. In this study monomicrobial and mixed polymicrobial biofilms were quantified using the XTT and CV methods [7,9].

### 3.1. XTT Method for the Quantification of Biofilms

**Figure 1:**
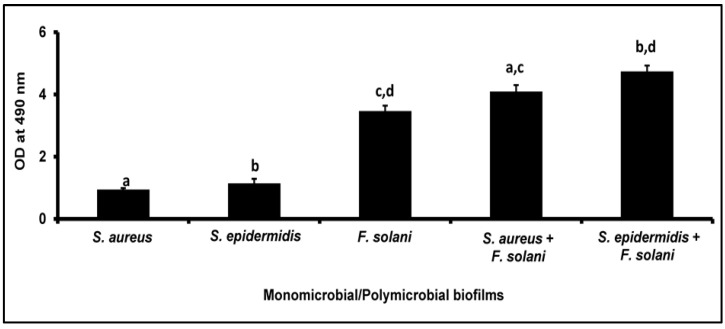
Quantification of the monomicrobial and mixed polymicrobial biofilms of ocular *S. aureus* and *S. epidermidis* with *F. solani* by the XTT method after 48 h of biofilm formation. Similar superscripts a, b, c, and d indicate significant increase (unpaired *t*-test, *p*-value < 0.05) in polymicrobial biofilm compared to the respective monomicrobial biofilm at 48 h. *S. aureus* ATCC 25923 was used as a positive control and *E. coli* ATCC 25922 was used as a negative control. Experiments were performed in triplicates. Values represent XTT absorbance at 490 nm expressed as average ± standard deviation.

Using the XTT method, it was observed that the monobacterial and monofungal biofilms of *S. aureus*, *S. epidermidis*, and *F. solani* increased at 48 h relative to 24 h (Table 1), thus confirming our earlier results in the monomicrobial biofilm of ocular pathogenic species such as *E. coli*, *S. aureus*, *S. epidermidis*, and *C. albicans* [8,9,46]. Further, when the mixed polymicrobial biofilms (*S. aureus* plus *F. solani* and *S. epidermidis* plus *F. solani*) were compared with corresponding monomicrobial biofilms at 48 h, the XTT value was significantly increased in the polymicrobial biofilm over the monomicrobial biofilms of both the bacterium and fungus involved (Table 1; Figure 1).

### 3.2. Crystal Violet Method for the Quantification of Biofilms

**Figure 2:**
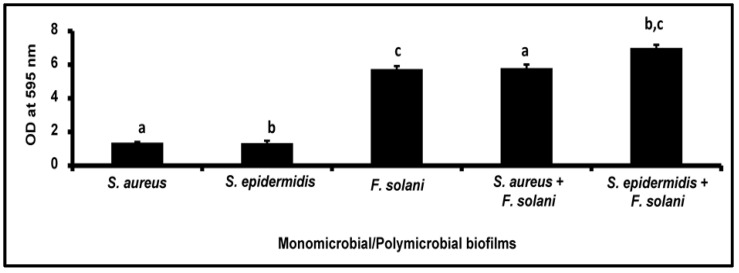
Quantification of the monomicrobial and mixed polymicrobial biofilms of *S. aureus* and *S. epidermidis* with *F. solani* by the CV method after 48 h of biofilm formation. Similar superscripts a, b, and c indicate significant increase (unpaired *t*-test, *p*-value < 0.05) in polymicrobial biofilm compared to the respective monomicrobial biofilm at 48 h. Experiments were performed in triplicate. Values represent CV absorbance at 595 nm expressed as average ± standard deviation.

The monomicrobial and polymicrobial biofilms showed an increase in the CV value in the biofilms at 48 h compared to the 24 h grown biofilm (Table 2). Further, the mixed polymicrobial biofilms showed an increase in the OD value of CV corresponding to either both or one of the components involved in the monomicrobial biofilm. For instance, the polymicrobial biofilm of involving *S. aureus* and *F. solani* showed an increase in the biofilm compared to only the bacterial component *S. aureus*, whereas *S. epidermidis* and *F. solani* showed an increase compared to *S. epidermidis* and *F. solani* monomicrobial biofilms (Table 2; Figure 2).

The discrepancy in the results between the XTT and CV methods could be because XTT measures the metabolic activity, whereas CV quantifies the biomass in the biofilm.

### 3.3. Confocal Laser Scanning Microscopy for Monitoring the Biofilm Thickness on Human Cadaveric Corneas

**Figure 3:**
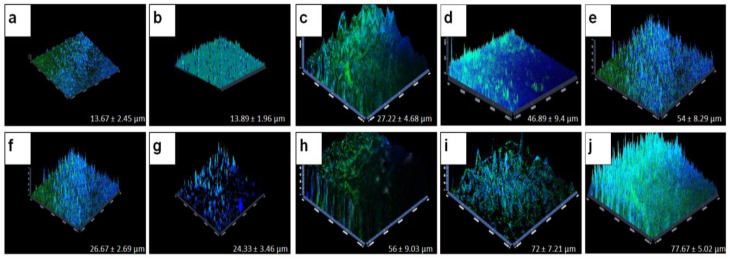
Biofilm thickness in monomicrobial and mixed polymicrobial biofilms of ocular *S. aureus*, *S. epidermidis*, and *F. solani* on human cadaveric corneas using confocal laser scanning microscope. Human cadaveric corneas were incubated to form monomicrobial and polymicrobial biofilms as indicated below for 24 and 48 h, respectively, at 37 °C in a 5% CO_2_ incubator. The formation of monomicrobial biofilms of the ocular bacteria and fungus are illustrated as follows: *S. aureus* biofilm at 24 h (**a**) and 48 h (**f**), *S. epidermidis* biofilm at 24 h (**b**) and 48 h (**g**), and *F. solani* biofilm at 24 h (**c**) and 48 h (**h**), respectively. The formation of the mixed polymicrobial biofilm of the ocular bacteria and fungus are illustrated as follows: mixed polymicrobial biofilms of *S. aureus* and *F. solani* at 24 h (**d**) and 48 h (**i**), and mixed polymicrobial biofilm of *S. epidermidis* and *F. solani* at 24 h (**e**) and 48 h (**j**). Biofilms were generated on cadaveric cornea as described in Materials and Methods and stained with Syto9^®^ and Calcofluor white. The fluorescent stains were excited at 490 nm and 363 nm, respectively. In the fluorescence micrographs, viable cells appear green in color and the extracellular polymeric substance appears blue in color. Images were taken at 40× magnification with zoom scale 2.

**Figure 4:**
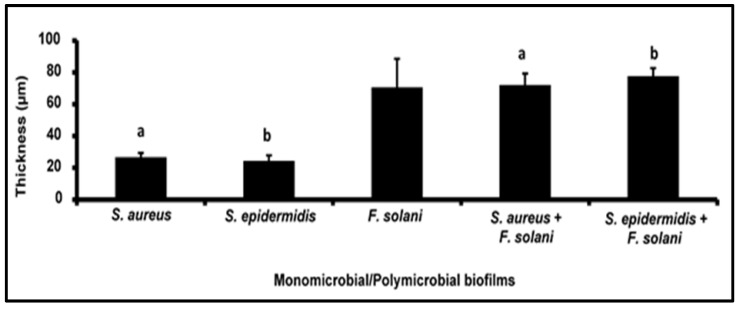
Biofilm thickness in monomicrobial and mixed polymicrobial biofilms on human cadaveric cornea using confocal laser scanning microscope. *S. aureus*, *S. epidermidis,* and *F. solani* monomicrobial biofilms at 48 h and mixed polymicrobial biofilms of *S. aureus* or *S. epidermidis* with *F. solani* at 48 h on human cadaveric corneas. Similar superscripts a and b indicate significant increase (unpaired *t*-test, *p*-value < 0.05) in mixed polymicrobial biofilm compared to the respective monomicrobial biofilm at 48 h. Experiments were performed in triplicates.

In addition to the CV and XTT methods which monitored the biofilm formation, the thickness of the biofilms formed by individual *S. aureus*, *S. epidermidis,* and *F. solani* and the corresponding polymicrobial biofilms of a bacterium with a fungus was also monitored by confocal laser scanning microscopy, which showed a distinct increase in the thickness at 48 h relative to the biofilms at 24 h (Table 3; Figure 3a,c,f,h). Further, the thickness of the mixed polymicrobial biofilms involving any two of the above taxa also increased at 48 h compared to the monomicrobial biofilms of either of the bacteria (*S. aureus* and *S. epidermidis*) but did not increase significantly in thickness compared to the biofilm formed by *F. solani* (Table 3; Figure 4). In Figure 4, it is clearly visible that the mixed polymicrobial biofilm was very thick and almost twice the thickness compared to the monomicrobial *S. aureus* and *S. epidermidis* individual biofilms (Table 3; Figure 4). For brevity, the thickness in the polymicrobial biofilms over the monobacterial biofilms is depicted only for 48 h of biofilm formation (Figure 4).

### 3.4. Scanning Electron Microscopy for Visualization of Biofilms on Human Cadaveric Cornea

**Figure 5:**
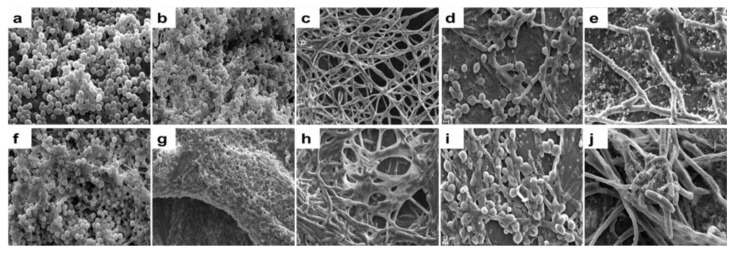
Visualization of monomicrobial and mixed polymicrobial biofilms involving ocular *S. aureus*, *S. epidermidis*, and *F. solani* on human cadaveric cornea using scanning electron microscopy. Human cadaveric corneas were incubated to form monomicrobial and polymicrobial biofilms as indicated below for 24 and 48 h at 37 °C in a 5% CO_2_ incubator. The formation of the monomicrobial biofilms of the ocular bacteria and fungus are illustrated as follows: *S. aureus* biofilm at 24 h (**a**) and 48 h (**f**), *S. epidermidis* biofilm at 24 h (**b**) and 48 h (**g**), *F. solani* biofilm at 24 h (**c**) and 48 h (**h**), respectively. The formation of the mixed polymicrobial biofilm of the ocular bacteria and fungus are illustrated as follows: mixed polymicrobial biofilm of *S. aureus* and *F. solani* at 24 h (**d**) and 48 h (**i**), mixed polymicrobial biofilm of *S. epidermidis* and *F. solani* at 24 h (**e**) and 48 h (**j**). Images were generated at 5000 × (**c**–**e**,**g**–**j**) and 10,000 × (**a**,**b**,**h**) magnification. Experiments were performed in triplicate.

Monomicrobial and polymicrobial biofilms were also monitored by scanning electron microscopy (SEM). *S. aureus*, *S. epidermidis,* and *F. solani* formed monomicrobial biofilms by 24 h (Figure 5a–c). By 48 h, multilayer clumps of cells were seen in the case of *S. aureus* and *F. solani*, whereas in *S. epidermidis*, the biofilm appeared as a huge column of multi-layered cells and exo polymeric substances (EPS) were clearly visible at 48 h relative to 24 h in the monomicrobial biofilms (Figure 5a–c,f–h). Further, when *S. aureus* was cultured together with *F. solani*, mixed polymicrobial biofilms were observed with clumps of *S. aureus* cells on *F. solani* (Figure 5d,i) and by 48 h, *S. aureus* were not easily visible and appeared to be enclosed in EPS (Figure 5d,i), implying that in the dual species, the interaction between the taxa may be influencing the biofilm process. Earlier, we had shown that ocular *S. aureus* and *S. epidermidis* formed mixed polymicrobial biofilms with *C. albicans* [9]. In the mixed polymicrobial biofilm involving *S. epidermidis* and *F. solani*, a clear association of the bacterium with the fungus was visible at 24 h, but by 48 h, the biofilm was very intense with an excessive amount of EPS, and only a few bacterial cells were visible on the biofilm (Figure 5e,j).

### 3.5. Susceptibility to Antimicrobials in Monomicrobial and Mixed Polymicrobial Biofilms Involving S. aureus and F. solani

*S. aureus* in the planktonic phase were resistant to all antibiotics except Amikacin and Chloramphenicol. The MIC of the antimicrobial corresponds to the concentration when none of the cells were viable and the OD at this concentration by the XTT method was <0.3 OD 490 nm. In *S. aureus*, the minimum biofilm eradication concentration (MBEC) of 18 antibiotics increased several folds (>2 fold) in the monomicrobial and mixed polymicrobial biofilms compared to the planktonic cells (Table 4). This is in accordance with our earlier studies on ocular *S. aureus* [8,51,58] which had exhibited several folds more resistance to all the antimicrobials tested compared to the planktonic cells. The susceptibility increased several folds (32 to >1024 µg/mL) both in the monomicrobial and mixed polymicrobial biofilms (Table 4) involving *S. aureus* and the fungus compared to the planktonic cells of *S. aureus* which exhibited most susceptibility to amikacin, ceftriaxone, ofloxacin, and chloramphenicol at 12 µg/mL. The mono- and mixed polymicrobial biofilms were least susceptible to azithromycin, metronidazole, and clindamycin (>1024 µg/mL) (Table 4). The MBEC was either increased with respect to 6 antibiotics, decreased with respect to 2 antibiotics, and showed no change to 10 antibiotics tested in the mixed polymicrobial biofilm compared to the individual biofilm of *S. aureus* (Table 4). This observation is in difference to earlier studies which indicated that mixed polymicrobial biofilms were more resistant to antimicrobials compared to the monomicrobial biofilms [59,60,61,62,63]. *S. aureus*–*F. solani* in the mixed polymicrobial biofilm (Table 4) showed an increase in resistance to the majority of aminoglycosides, one fluoroquinolone (ciprofloxacin), one amphenicol (chloramphenicol), and one tetracycline antibiotic (Table 4) compared to the monomicrobial biofilms. A few reports also indicated that in polymicrobial biofilms, *C. albicans* enhanced the resistance of *S. aureus* [63,64,65] to daptomycin and vancomycin and several other antibiotics as in this study [9,12]. The increase in the MBEC has been attributed to poor antibiotic penetration, nutrient limitation, slow growth, stress, the formation of persister cells, and the formation of an extracellular biofilm matrix [12,66,67,68]. Trizna et al. (2020) had earlier demonstrated that in *S. aureus* plus *P. aeruginosa* dual species biofilms, a ten-fold increase in the susceptibility to ciprofloxacin and aminoglycosides (gentamicin or amikacin) was observed compared to monobacterial biofilms. Interestingly, in this study, both the mixed polymicrobial biofilms (bacterium plus fungus) also showed a decrease in resistance to gatifloxacin and moxifloxacin (Table 4). This contradicts earlier studies which indicated that polymicrobial biofilms are more challenging to treat since they are more resistant to antimicrobial treatment than the corresponding single-species biofilms [5,9,58,59].

### 3.6. Susceptibility to Antimicrobials in Monomicrobial and Mixed Polymicrobial Biofilms Involving S. epidermidis and F. solani

The MBEC of *S. epidermidis* in the monomicrobial and polymicrobial biofilm phases increased several folds (>2 fold) compared to the planktonic cells for all the 18 different antibiotics that were screened (Table 5). Similar results were reported earlier, which indicated that *S. epidermidis* in the biofilm phase were more resistance to antimicrobials compared to the planktonic phase [12,69].

*S. epidermidis* in the planktonic phase were resistant to all antibiotics except Amikacin and Chloramphenicol. and was most susceptible to Amikacin, ceftriaxone, and cefazolin at 12 µg/mL, which increased to 480 to >1024 µg/mL both in the monomicrobial and mixed polymicrobial biofilm (Table 5). It was also observed that the mono- and polymicrobial biofilms were least susceptible to azithromycin, metronidazole, and clindamycin (>1024 µg/mL) (Table 5). However, when we compared the MBEC between the monomicrobial and polymicrobial biofilms of *S. epidermidis* and *F. solani* in the polymicrobial biofilms, the MBEC decreased in six, increased in five, and the remaining five antibiotics did not show any change in the MBEC (Table 5). *S. epidermidis* plus *F. solani* showed an increase in the MBEC only with respect to three of the four cephalosporins that were screened. In cases of cystic fibrosis, *P. aeruginosa* and *Inquilinus limosus or Dolosigranulum pigrum* showed an increase in resistance to most antibiotics [70].

Decreased resistance to few antibiotics observed in polymicrobial biofilms (*S. aureus* + *F. solani*) and (*S. epidermidis* + *F. solani*) compared to the respective mono-bacterial biofilms probably implied that the biotic components (bacteria and fungi) within the biofilm were interacting and making them more sensitive to the drugs.

### 3.7. Susceptibility of F. solani in Monomicrobial (F. solani) and Mixed Polymicrobial Biofilms (F. solani plus S. aureus; F. solani plus S. epidermidis) with Planktonic Cells of F. solani

*F. solani* in the biofilm phase also showed a reduced susceptibility to the antifungals compared to the planktonic cells of *F. solani* (Table 6), similar to earlier reports on ocular *C. albicans* biofilms [8]. The mixed polymicrobial biofilms also showed an increase in the MBEC compared to the planktonic cells of *F. solani*. However, the mixed polymicrobial biofilms of *F. solani* with either of the bacterium showed either an increase, decrease, or no change in the MBEC compared to the monomicrobial *F. solani* biofilm for the antifungals (Table 6). The results mimic the observations of the above two bacteria with the fungus *C. albicans* [9]. Earlier studies observed that *S. epidermidis* protects *C. albicans* from the action of the antifungal drugs fluconazole and amphotericin B in polymicrobial biofilms [66], thus making them more resistant. This is further complicated by the protective effect of the biofilms, virulence enhancement, and horizontal gene transfer in the biofilm [71].

### 3.8. Limitations and Relevance of the Study

Similar studies involving other bacterial and fungal etiological agents of ocular disease may help in a more directed ocular disease treatment. This study does not unravel the molecular basis for the formation of polymicrobial biofilms with respect to the individual taxa involved and the reasons for the differential susceptibility to antimicrobials in the polymicrobial biofilm phase.

To the best of our knowledge, this is the first study demonstrating that ocular bacteria and a filamentous fungi possess the potential to form monomicrobial biofilms which exhibit an increased resistance to both antibacterial and antifungal agents compared to planktonic cells. Further, the mixed polymicrobial biofilms showed either an increase or decrease in resistance compared to the monomicrobial biofilms and, surprisingly, in many cases, the mixed polymicrobial biofilms did not show any change in the resistance to antimicrobials.

## 4. Conclusions

In the present study, we demonstrated that the ocular pathogens of *S. aureus*, *S. epidermidis*, and *F. solani* form monomicrobial and mixed polymicrobial biofilms both on tissue culture polystyrene plates and on ex vivo human corneas from cadavers using a confocal microscope and a scanning electron microscope. Further, in the biofilm phase, monomicrobial species and mixed polymicrobial species were several times more resistant to antibacterial and antifungals agents, respectively, compared to planktonic phase cells. Additionally, mixed polymicrobial species exhibited either an increase, decrease, or no change in the MBEC to antimicrobials compared to the monomicrobial biofilms. These studies would be very relevant in planning treatment strategies for preventing biofilm formation and overcoming drug resistance in eye infections.

## Figures and Tables

**Table 1:** Formation of monomicrobial and polymicrobial biofilms by ocular *S. aureus*, *S. epidermidis*, and *F. solani* by the XTT method.

S. No.	Microorganism	XTT OD _490 nm_ after 24 h of Biofilm Formation	XTT OD _490 nm_ after 48 h of Biofilm Formation *
Monomicrobial biofilm			
1	*S. aureus* L1054/2020(2)	0.46 ± 0.1	0.94 ± 0.04 ^a^
2	*S. epidermidis* L1058/2020(2)	0.74 ± 0.13	1.14 ± 0.14 ^b^
3	*F. solani* (L1579/2020)	2.62 ± 0.06	3.46 ± 0.18 ^c,d^
Mixed polymicrobial biofilm			
4	*S. aureus* + *F. solani*	3.42 ± 0.17	4.09 ± 0.21 ^a,c^
5	*S. epidermidis + F. solani*	3.61 ± 0.36	4.74 ± 0.19 ^b,d^
Control			
6	*S. aureus* ATCC 25923 (positive control)	0.66 ± 0.015	1.35 ± 0.095
7	*E. coli* ATCC 25922 (negative control)	0.17 ± 0.026	0.27 ± 0.06

* OD of YPD broth without inoculum (no organism control) was deducted from the OD of monomicrobial/polymicrobial biofilms. Similar superscripts ^a^, ^b^, ^c^, and ^d^ indicate that the XTT ODs are significantly different (*p*-value < 0.05) between the indicated rows. Experiments were performed in triplicate.

**Table 2:** Formation of monomicrobial and polymicrobial biofilms by ocular *S. aureus*, *S. epidermidis*, and *F. solani* by the crystal violet method *.

S. No.	Microorganism	CV OD _595 nm_ after 24 h of Biofilm Formation *	CV OD _595 nm_ after 48 h of Biofilm Formation *
Monomicrobial biofilm			
1	*S. aureus* L1054/2020(2)	0.82 ± 0.06	1.36 ± 0.13 ^a^
2	*S. epidermidis* L1058/2020(2)	0.87 ± 0.08	1.33 ± 0.07 ^b^
3	*F. solani* (L1579/2020)	4.29 ± 0.31	5.74 ± 0.44 ^c^
Mixed polymicrobial biofilm			
4	*S. aureus* + *F. solani*	4.25 ± 0.11	5.8 ± 0.49 ^a^
5	*S. epidermidis + F. solani*	4.47 ± 0.29	6.99 ± 0.15 ^b,c^
Control			
6	*S. aureus* ATCC 25923 (positive control)	1.36 ± 0.10	1.98 ± 0.30
7	*E. coli* ATCC 25922 (negative control)	0.15 ± 0.10	0.35 ± 0.30

* OD of YPD broth without inoculum (no organism control) was deducted from the OD of monomicrobial/polymicrobial biofilms. Similar superscripts ^a^, ^b^, and ^c^ indicate values are significantly different (*p*-value < 0.05) between the indicated rows. Experiments were performed in triplicate.

**Table 3:** Biofilm thickness of monomicrobial and mixed polymicrobial biofilms of ocular *S. aureus*, *S. epidermidis*, and *F. solani* on cadaveric human cornea by confocal laser scanning microscopy.

S. No.	Microorganism	Thickness in µm after 24 h of Biofilm Formation	Thickness in µm after 48 h of Biofilm Formation *
Monomicrobial biofilm			
1	*S. aureus* L1054/2020(2)	13.67 ± 2.45	26.67 ± 2.69 ^a^
2	*S. epidermidis* L1058/2020(2)	13.89 ± 1.96	24.33 ± 3.46 ^b^
3	*F. solani* (L1579/2020)	27.22 ± 4.68	56 ± 9.03
Mixed polymicrobial biofilm			
4	*S. aureus* + *F. solani*	46.89 ± 9.4	72 ± 7.21 ^a^
5	*S. epidermidis +* *F. solani*	54 ± 8.29	77.67 ± 5.02 ^b^

* Similar superscripts ^a^ and ^b^ indicate that the values are significantly different (*p*-value < 0.05 by *t*-test) between the indicated rows. Experiments were performed in triplicate.

**Table 4:** Determination of MBEC * of antibiotics in the monomicrobial and mixed polymicrobial biofilms of *S. aureus* compared to MIC ^#^ in planktonic bacterial cells using the XTT method (OD 490 nm).

	Antibiotics (µg/mL)	*S. aureus* Planktonic Phase (48 h) (MIC ^#^)	*S. aureus* Biofilm Phase(48 h) (MBEC *)	*S. aureus* + *F. solani* Biofilm Phase (48 h) (MBEC *)
Aminoglycosides	Amikacin	12 (S)	512	1024
	Gentamicin	24 ^®^	480	1024
	Tobramycin	24	256	512
β-lactam	Ampicillin	24	256	256
Cephalosporins	Cefuroxime	24	512	512
	Ceftriaxone	12	512	512
	Cefepime	48	1024	1024
	Cefazolin	24	480	480
Fluoroquinolones	Gatifloxacin	20 (R)	>1024	1024
	Moxifloxacin	48 (R)	>1024	1024
	Ciprofloxacin	24 (R)	128	512
	Ofloxacin	12 (R)	512	512
Amphenicols	Chloramphenicol	12 (S)	32	128
Macrolide	Azithromycin	48 (R)	>1024	>1024
Nitroimidazole	Metronidazole	24	>1024	>1024
Lincosamide	Clindamycin	48 (R)	>1024	>1024
	Lincomycin	24 (R)	512	512
Tetracycline	Monocycline	24 (R)	64	128

* MBEC, minimum biofilm eradication concentration (µg/mL); ^#^ MIC, minimum inhibitory concentration (µg/mL); R, resistant; S, sensitive. R and S concentrations based on CLSI guidelines (CLSI/NCCLS Document M100-S22. Wayne, PA: Clinical and Laboratory Standards Institute, 2012). Break points are not available for Tobramycin, Ampicillin, Cefuroxime, Ceftriaxone, Cefepime, Cefazolin, and Metronidazole in CLSI guidelines. All experiments were carried out thrice.

**Table 5:** Determination of MBEC * of antibiotics in the monomicrobial and mixed polymicrobial biofilms of *S. epidermidis* compared to MIC ^#^ in planktonic bacterial cells using the XTT method (OD 490 nm).

	Antibiotics (µg/mL)	*S. epidermidis* Planktonic Phase (48 h) (MIC ^#^)	*S. epidermidis* Biofilm Phase (48 h) (MBEC *)	*S. epidermidis* + *F. solani* Biofilm Phase (48 h) (MBEC *)
Aminoglycosides	Amikacin	12 (S)	>1024	1024
	Gentamicin	24 (R)	1024	512
	Tobramycin	48	256	256
β-lactam	Ampicillin	48	>1024	1024
Cephalosporins	Cefuroxime	24	512	1024
	Ceftriaxone	12	512	1024
	Cefepime	48	>1024	1024
	Cefazolin	12	480	1024
Fluoroquinolones	Gatifloxacin	20 (R)	>1024	1024
	Moxifloxacin	48 (R)	>1024	1024
	Ciprofloxacin	24 (R)	128	64
	Ofloxacin	32 (R)	1024	1024
Amphenicols	Chloramphenicol	20 (S)	128	256
Macrolide	Azithromycin	128 (R)	>1024	>1024
Nitroimidazole	Metronidazole	24	>1024	>1024
Lincosamide	Clindamycin	48 (R)	>1024	>1024
	Lincomycin	32 (R)	1024	512
Tetracycline	Monocycline	20 (R)	256	512

* MBEC—minimum biofilm eradication concentration (µg/mL); ^#^ MIC—minimum inhibitory concentration (µg/mL); R, resistant; S, sensitive. R and S concentrations based on CLSI guidelines (CLSI/NCCLS Document M100-S22. Wayne, PA: Clinical and Laboratory Standards Institute, 2012). Break points are not available for Tobramycin, Ampicillin, Cefuroxime, Ceftriaxone, Cefepime, Cefazolin, and Metronidazole in CLSI guidelines. All experiments were carried out thrice.

**Table 6:** Determination of MBEC * of antifungal agents for *F. solani* in a monomicrobial (*F. solani*) and mixed polymicrobial biofilms (*S. epidermidis* or *S. aureus* plus *F. solani*) compared to MIC ^#^ in planktonic fungal cells using the XTT method (OD 490 nm).

Antifungal (µg/mL)	*F. solani* Planktonic Phase (48 h) ^#^ (MIC)	*F. solani* Biofilm Phase (48 h) * (MBEC)	*F. solani* + *S. aureus* Biofilm Phase (48 h) * (MBEC)	*F. solani* + *S. epidermidis* Biofilm Phase (48 h) * (MBEC)
Amphotericin B	1	48	48	48
Caspofungin	2	48	64	128
Fluconazole	32	512	256	512
Itraconazole	32	512	128	256
Natamycin	8	64	64	80
Voriconazole	4	64	64	128

* MBEC—minimum biofilm eradication concentration (µg/mL); ^#^ MIC—minimum inhibitory concentration (µg/mL). All experiments were carried out thrice. Break points are not available for *F. solani* in CLSI guidelines (CLSI/NCCLS Document M100-S22. Wayne, PA: Clinical and Laboratory Standards Institute, 2012).

## Data Availability

Not applicable.
